# Combined Use of Ibalizumab and Lenacapavir for the Treatment of Multidrug-resistant HIV-1: A Case Series

**DOI:** 10.1093/cid/ciaf597

**Published:** 2025-11-03

**Authors:** Charlotte Rolle, Amit Achhra, Christina Harbison, Diana Finkel, Christopher Lucasti, Claudia Martorell, Tammie McClendon, Jihad Slim

**Affiliations:** Research Operations, Orlando Immunology Center, Orlando, Florida, USA; Infectious Diseases, Yale School of Medicine, New Haven, Connecticut, USA; Clinical Pharmacy, Prism Health North Texas, Dallas, Texas, USA; Rutgers Health, New Jersey Medical School, Newark, New Jersey, USA; Shore Medical Center, South Jersey Infectious Disease, Somers Point, New Jersey, USA; Clinical/Medical and Research Divisions, The Research Institute, Springfield, Massachusetts, USA; Infectious Diseases, Wayne Health, Detroit, Michigan, USA; School of Medicine, New York Medical College, Valhalla, New York, USA

**Keywords:** heavily treatment-experienced persons with HIV, ibalizumab, injectable antiretroviral, lenacapavir, multidrug-resistant HIV-1

## Abstract

**Background:**

Some individuals with human immunodeficiency virus (HIV-1) have acquired multidrug-resistant (MDR) strains of HIV and/or are nonadherent to antiretroviral (ARV) medication. Injectable ARVs can provide salvage therapy for those with limited therapeutic options and may be preferred by some people with HIV (PWH). Real-world evidence may contribute to a more comprehensive understanding of the barriers to adherence and the utility of injectable ARVs in PWH. Currently, there is a lack of data on combined use of injectable ibalizumab (IBA) and lenacapavir (LEN) with optimized background regimen (OBR).

**Methods:**

A retrospective observational study examined medical charts from people with MDR HIV across 8 facilities in the United States. All PWH used a combination of both IBA + LEN ± OBR for at least 6 months. Viral loads and CD4^+^ counts were collected.

**Results:**

A total of 21 PWH were included. Four-class resistance at baseline was reported in 38.1% of PWH. Within 12 to 24 weeks of combined IBA + LEN treatment, a median reduction of −2710 copies/mL HIV RNA was observed. Median increase to CD4^+^ count was 67.5 cells/mm^3^ within 4 to 44 weeks of treatment initiation. Few intolerances required changes to treatment. Therapy with IBA + LEN continued for an average of 30 months and 20 months, respectively.

**Conclusions:**

In this small group of individuals with MDR HIV who were heavily treatment-experienced and/or faced adherence challenges, the use of IBA + LEN ± OBR was well tolerated and led to clinically significant reductions in viral loads and improvements in CD4^+^ counts.

## Teaser

A group of 21 individuals living with multidrug-resistant HIV and/or adherence challenges received injectable ibalizumab and lenacapavir plus optimized background regimen for at least 6 months. People with HIV experienced clinically significant reductions in viral load and increases in CD4^+^ count after combined treatment.

**(See the Editorial Commentary by Short and Ogbuagu on pages e541–3.)**

Recent data indicate that less than 3% of people with human immunodeficiency virus (HIV-1; PWH) in the United States are diagnosed with viral isolates resistant to 2 or more classes of antiretroviral (ARV) medications [1, 2], illustrating a relatively low rate of transmitted HIV resistance. Treatment-experienced PWH demonstrate higher levels of cumulative acquired resistance; for example, in a US population of 489 Black PWH with genotypic data available, 9% and 3% of individuals were diagnosed with isolates resistant to 2 and 3 classes, respectively [3]. Although this subgroup of PWH is small, there is still an urgent need for treatment alternatives in PWH with multidrug-resistant (MDR) HIV who may be heavily treatment-experienced (HTE) or have limited treatment options [4–6]. It is known that people with MDR HIV are at risk of poorer outcomes than those not experiencing high levels of resistance [7], and that lack of adherence to ARVs can lead to subtherapeutic drug levels, incomplete suppression of viral replication, and selection for resistance [5]. The social determinants of health can play a large role in someone's ability to adhere to ARVs, and thus in viral suppression [5, 8, 9].

There are limited therapeutic options for people with MDR HIV, and so some choose injectable ARVs out of necessity for a salvage regimen, whereas others choose this route based on personal preference [10–12]. PWH without resistance challenges may also choose injectable formulations because of the risk of drug–drug interactions (DDIs) with specific oral agents, the experience of side effects with oral ARVs, or other factors such as comorbidities. Some PWH state they prefer injectables because of scheduling convenience, ensuring treatment confidentiality, avoiding daily reminders of HIV status, or the belief that injections are more effective than oral treatments [13, 14]. Preference for injections is individualized, and we must understand each person's perspective. For some PWH, use of long-acting injectable ARVs can help to improve adherence [15]. However, HIV drug resistance can preclude certain individuals from accessing even the fully injectable regimen of cabotegravir (CAB) and rilpivirine (RPV) because this regimen is challenged by isolates with full or partial resistance and, therefore, not suitable for all PWH [15, 16]. Patient selection models for CAB + RPV regimens examine baseline factors to help clinicians determine those PWH most likely to experience confirmed virologic failure [17].

An undetectable viral load is still the treatment goal for most PWH but achieving it is not always simple or feasible. For some PWH, reducing viral load (VL) and increasing CD4^+^ T-cell count may be an achievable step to improving their quality of life and/or life expectancy [5].

ARVs with novel mechanisms of action are approved for people with MDR HIV who are HTE [18]. Ibalizumab (IBA) is a humanized immunoglobulin G4 monoclonal antibody that binds to domain 2 of the CD4 receptor to prevent conformational changes in gp120, which is necessary for viral entry [19]. After a loading dose, IBA is administered by intravenous injection (infusion or push) every 2 weeks [20]. Lenacapavir (LEN) is a capsid inhibitor that interferes with several stages of the viral life cycle to inhibit viral replication [21]. LEN requires an oral loading dose followed by subcutaneous injection every 6 months [22]. Although both agents have been tested in randomized trials, there are limited real-world data on combined use of IBA + LEN.

The objective of this case series is to demonstrate real-world outcomes of combined use of IBA + LEN ± optimized background regimen (OBR) in HTE patients with MDR HIV. These outcomes may inform clinicians on the utility of injectable regimens.

## METHODS

This is a retrospective case series review following CARE case report guidelines. A waiver for patient consent procedures was issued by Advarra Institutional Review Board after full examination of the protocol; therefore, patients did not provide written consent to investigators (see Supplementary, IRB exemption).

Eight clinician investigators from different treatment facilities in the United States (Connecticut, Florida, Massachusetts, Michigan, New Jersey, New York, and Texas) reviewed patient charts to identify PWH who were prescribed both IBA + LEN. Charts were screened to determine which patients had combined use of both drugs for ≥6 months and had measurement of VL before treatment initiation with follow-up measurement by 6 months after initiation. Patients were required to have a diagnosis of MDR HIV, documented issues with DDIs, or documented intolerance to oral ARVs. Standardized case collection forms developed by the investigators were used to obtain patient demographics and clinical characteristics, prior ARV use, VL and CD4^+^ counts, summaries of resistance testing, therapies included in the OBR, reported side effects or intolerances, adherence barriers documented in the clinical record, and clinician-perceived benefit to the patient. Clinicians provided a summary of resistance testing for each patient rather than a comprehensive list of International Antiviral Society-United States of America–designated mutations. Patient numbers were assigned to clinicians for them to maintain control of all confidential patient information. Deidentified patient information was collected on the numbered forms (see Supplementary, Case collection form) and collated by an external medical writer while being stored on a password-protected server. Patient confidentiality was maintained at all stages of data collection, analysis, and manuscript development.

Categorical changes to VL and CD4^+^ count compared to baseline were analyzed using a Fisher exact *t*-test, and types of reported side effects or adverse events were tabulated. VL was categorized according to the following classification: <50 copies/mL; 50 to <200 copies/mL; 200 to <400 copies/mL; and ≥400 copies/mL. CD4^+^ counts were categorized as: <200 cells/mm^3^; 200 to <350 cells/mm^3^; 350 to <500 cells/mm^3^; and ≥500 cells/mm^3^. Statistical summaries include sample size (n), mean, median, range, and percentage. Analysis of safety and tolerability is descriptive. Clinicians had the opportunity to provide their overall opinion on the benefits of combined IBA + LEN to the patient using a 4-point Likert scale (Significant benefit, Moderate benefit, Some benefit, No benefit). They could also provide an informal text description on how they perceived the impact of combined treatment on the patient. Because of the retrospective nature of the data collection, timing of patient visits was not standardized across the group.

## RESULTS

### Baseline Characteristics

The total number of patients identified was 23; however, 2 were excluded because the length of combined treatment with IBA + LEN was less than 6 months. Baseline characteristics of the 21 patients included in this analysis are shown in Table 1.

**Table 1.** Baseline Characteristics

Characteristic	N = 21
Mean age in years (range)	51 (29–74)^a^
Sex, n (%)	
Male	15 (71%)
Race and ethnicity, n (%)	
White/Caucasian	8 (38.1)
Black/African American	12 (57.1)
Unknown	1 (4.8)
Hispanic	4 (19.0)
Non-Hispanic	7 (33.3)
Not reported	10 (47.6)
Mean time since diagnosis in years (range)	28 (3–40)^a^
Baseline VL (copies/mL)	
Mean	160 650
Median	5350
Range	<20–1 200 000
Baseline CD4^+^ count (cells/mm^3^)	
Mean	254
Median	257
Range	<20–609
Barriers to ARV adherence^b^, n	
Multiple doses of oral medication each day	5
Memory issue	4
Access issue	4
Lack of family/social support	3
Medication beliefs	2
HIV status disclosure issue	2
Pill fatigue or burden	2
Nausea or GI intolerance to oral meds	2
Swallowing issue/feeding tube	2
Not reported	8
Resistance pattern, n (%)	
One-class	1 (4.8)
Two-class	6 (28.5)
Three-class	5 (23.8)
Four-class	8 (38.1)
Not available (VL too low for testing)	1 (4.8)
Resistance to drug classes, n^c^ (%)	
NRTI	19 (95)
NNRTI	14 (70)
PI	12 (60)
INSTI	15 (75)

Abbreviations: ARV, antiretroviral; GI, gastrointestinal; INSTI, integrase strand transfer inhibitor; NNRTI, non-nucleoside reverse transcriptase inhibitor; NRTI, nucleoside reverse transcriptase inhibitor; PI, protease inhibitor; VL, viral load.

^a^One person's age was not provided; n = 20.

^b^Records may have indicated more than 1 barrier to adherence.

^c^One patient's resistance testing was not provided as viral loads were too low for evaluation; n = 20.

Mean (range) patient age was 51 (29–74) years; 71% were male and there was a diverse mix of races and ethnicities. The average time since diagnosis was 28 years. Resistance testing indicated the following rates of resistance present at baseline or from archived information: 95% of patients had resistance to nucleoside reverse transcriptase inhibitor (NRTI), 70% to nonnucleoside reverse transcriptase inhibitor (NNRTI), 60% to protease inhibitor, and 75% to integrase strand transfer inhibitor. Most patients (38.1%) had 4-class resistance (Table 1). Before initiation of IBA + LEN, patients had a median (range) VL of 5350 (<20–1 200 000) copies/mL and a median (range) CD4^+^ cell count of 257 (<20–608) cells/mm^3^.

There were many factors that clinicians documented as potential barriers to ARV adherence. The most commonly reported barrier to adherence was having to take multiple doses of oral medication each day. However, other factors included issues with memory, lack of social or family support, adverse gastrointestinal effects of oral medications, difficulty swallowing pills, pill fatigue, or needing to administer oral medications through feeding tubes (Table 1). Clinicians also noted comorbidity (eg, depression, anxiety), history of alcohol or substance use, and unstable housing or financial support. One patient initiated IBA + LEN to avoid a potential DDI with their chemotherapy regimen.

Patients had a complex history of prior ARV use. A listing of ARVs used immediately before initiation of IBA + LEN, OBR during treatment, and subsequent changes to regimens can be found in Supplementary Table 1. It is notable that, with initiation of IBA + LEN, 8/21 patients (38.0%) were on fully injectable regimens. Four of 21 patients (19.0%) did not have any OBR agents used in conjunction with IBA + LEN, and 4/21 patients (19.0%) used a regimen of CAB/RPV with IBA + LEN. Changes to OBR during treatment occurred in 4 patients (19.0%), with 3 patients (14.3%) simplifying treatment and 1 patient (4.8%) requiring the addition of an oral ARV. A total of 12/21 patients (57.1%) received IBA injections at home at some point during the observation period (Supplementary Table 1).

### Real-world Effectiveness

**Figure 1.**
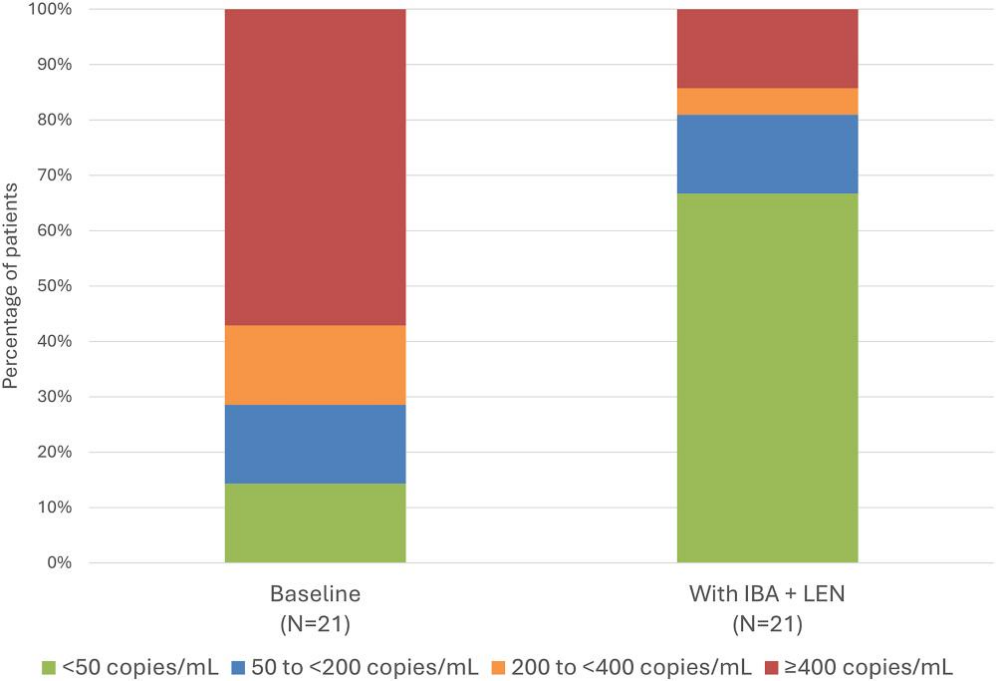
Changes to VL after combined IBA + LEN treatment (± OBR). VL was measured 12–24 wks after the combined use of IBA + LEN. *P* = .0023 according to Fisher exact *t*-test. Abbreviations: IBA, ibalizumab; LEN, lenacapavir; OBR, optimized background regimen; VL, viral load.

Changes in VL from baseline were variable across the group of patients; however, 9/21 patients (42.8%) had a VL ≥200 copies/mL at baseline and achieved <50 copies/mL within 12 to 24 weeks of combined use of IBA + LEN (Table 2). By the end of this observation period, 14/21 patients (66.6%) had a VL <50 copies/mL (Figure 1) and only 1 patient experienced an increase in VL (change from 200 to <400 copies/mL at baseline to ≥400 copies/mL at follow-up). No patients who were undetectable (VL <50 copies/mL) at baseline experienced an increase above 50 copies/mL after IBA + LEN treatment. Median change from baseline in VL was −2710 (or −3.43 log_10_) copies/mL (mean change: −159 860 copies/mL; range: −1 199 887 to 552; n = 21).

**Table 2.** Outcomes of Treatment

Parameter, n (%)	N = 21
Virologic outcomes: Changes to VL	
<50 copies/mL at baseline and maintained	3 (14.3)
50 to <200 copies/mL at baseline and decreased to <50 at follow-up	2 (9.5)
200 to <400 copies/mL at baseline and decreased to <50 at follow-up	2 (9.5)
≥400 copies/mL at baseline and decreased to <50 at follow-up	7 (33.3)
50 to <200 copies/mL at baseline and maintained	1 (4.8)
50 to <200 copies/mL at baseline and increased to 200 to <400 at follow-up	0
200 to <400 copies/mL at baseline and increased to ≥400 at follow-up	1 (4.8)
≥400 copies/mL at baseline and maintained	2 (9.5)
≥400 copies/mL at baseline and decreased to 50 to <200 at follow-up	2 (9.5)
≥400 copies/mL at baseline and decreased to 200 to <400 at follow-up	1 (4.8)
Immune status: Changes to CD4^+^ count	
<200 cells/mm^3^ at baseline and remained <200 at follow-up	5 (23.8)
<200 cells/mm^3^ at baseline and increased to 200 to <350 at follow-up	2 (9.5)
<200 cells/mm^3^ at baseline and increased to 350 to <500 at follow-up	2 (9.5)
200 to <350 cells/mm^3^ at baseline and maintained	2 (9.5)
200 to <350 cells/mm^3^ at baseline and decreased to <200 at follow-up	1 (4.8)
350 to <500 cells/mm^3^ at baseline and maintained	1 (4.8)
350 to <500 cells/mm^3^ at baseline and decreased to 200 to <350 at follow-up	1 (4.8)
350 to <500 cells/mm^3^ at baseline and increased to ≥500 at follow-up	1 (4.8)
≥500 cells/mm^3^ at baseline and remained ≥500 at follow-up	3 (14.3)
Changes not evaluable	3 (14.3)

Follow-up periods varied, but changes to viral load were reported within a range of 12 to 24 wks after treatment initiation. Changes to CD4^+^ count were reported within a range of 4 to 44 wks.

Abbreviation: VL, viral load.

Changes to CD4+ count compared with baseline were highly variable. Of the 18 patients with evaluable changes, 14 (77.8%) experienced some increase in CD4^+^ count during the observation period; however, these changes may not have been clinically significant in all cases (Supplementary Table 1). There were 4 patients (22.2%) with CD4^+^ count <200 cells/mm^3^ at baseline who achieved ≥200 cell/mm^3^ at follow-up (Table 2). Follow-up time for CD4^+^ counts varied from 4 to 44 weeks after initiation of combined IBA + LEN; these could not be standardized because of retrospective data collection. Median change from baseline in CD4^+^ count was 67.5 cells/mm^3^ (mean change: 73.8 cells/mm^3^; range: −106 to 275; n = 18).

Clinicians were asked to report their perspectives on patient benefit of combined use of IBA + LEN. Clinicians reported significant benefit in 7/21 patients (33.3%), although it should be noted that a clinician response was not provided for 8/21 patients (38.1%; Table 3). One patient achieved undetectable viral loads and so was eligible for biologic therapy for an autoimmune disease. In some patients, combined treatment with IBA + LEN provided the greatest reductions in VL and/or increases in CD4^+^ count that the clinician had seen in several years of follow-up with those patients.

**Table 3.** Clinician Perception of IBA + LEN Benefit to Patient

Benefit, n (%)	N = 21
Significant benefit	7 (33.3)
Moderate benefit	4 (19.0)
Some benefit	2 (9.5)
No benefit	0
Not reported	8 (38.1)

Clinicians rated their perception of patient benefit according to a 4-point Likert scale.

Abbreviations: IBA, ibalizumab; LEN, lenacapavir.

### Safety and Tolerability

The mean (range) duration of treatment with IBA was 30 (9–72) months and 20 (9–56) months with LEN. Most of the reported intolerances or side effects were mild or self-limiting and did not require changes to treatment (Table 4).

**Table 4.** Side Effects or Intolerances Reported During Treatment

Side Effect/Intolerance Reported, n (%)	N = 21
Cough	1 (4.8)
Tenderness at LEN injection sites (nodules ∼10 cm diameter)	1 (4.8)
Mild injection site discomfort/pain	3 (14.3)
Fever, chills, nausea, diarrhea	1 (4.8)
Flushing, itchiness, rash, diarrhea	1 (4.8)
Changes to clotting (not specified)	1 (4.8)^a^

Abbreviation: LEN, lenacapavir.

^a^Ibalizumab was discontinued because of that clinician's concern that it was affecting clotting.

**Figure 2.**
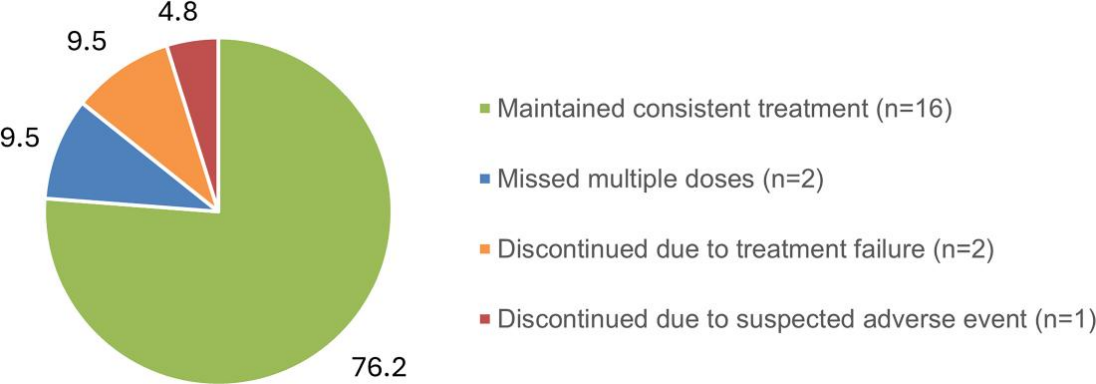
Patient disposition by end of observation period (N = 21) (%). A suspected adverse event that required treatment discontinuation occurred in one patient. IBA was discontinued because of a concern that it was affecting clotting. Treatment failure was defined by the clinician and noted on the record. Missed multiple doses were due to nonadherence, hospitalization, or scheduling issues. Abbreviation: IBA, ibalizumab.

IBA was discontinued in 1 patient because of a concern that it was affecting clotting. Two patients discontinued treatment because of reported treatment failure (Figure 2). Treatment failure was defined by the clinician and documented on the patient record. The 2 patients who experienced treatment failure were viremic at baseline (VL ≥400 copies/mL) and remained detectable during combined IBA + LEN treatment. Both patients had 2-class resistance at baseline; 1 patient had resistance to NRTI and integrase strand transfer inhibitor, and the other patient had resistance to NRTI and NNRTI. In both cases, IBA + LEN was discontinued by the clinician. Further resistance testing obtained for the former patient showed that the strain contained Q67H and K70R mutations, both of which are associated with reduced susceptibility to LEN. It is unknown if the Q67H and K70R mutations were present at baseline. Based on an assay from Monogram Biosciences, there was some resistance to IBA noted, although details were limited. A full listing of strain mutations for this patient (patient 8) can be found in Supplementary Table 1.

Two patients missed multiple doses of IBA and/or LEN because of nonadherence, hospitalization, or scheduling issues. A few patients reported injection fatigue but still continued to receive 100% of their scheduled doses. At the end of the observation period, 16/21 patients (76.2%) maintained consistent treatment with IBA + LEN (Figure 2).

## DISCUSSION

In this case series of HTE PWH with high levels of multiclass resistance, combined use of IBA + LEN ± OBR was well tolerated and resulted in clinically significant changes to VL, as well as improvements in CD4^+^ count. Median change from baseline in VL was −2710 copies/mL; median change from baseline in CD4^+^ count was 67.5 cells/mm^3^. Most promising was the percentage of patients (42.8%) with baseline VL >200 or 400 copies/mL who achieved VL <50 copies/mL after treatment with combined IBA + LEN. For some of these patients, the treatment provided an extended period of viral suppression that had not been achieved in years of follow-up. Likewise, increases in CD4^+^ count were noted across most patients, although it was not always possible to evaluate the clinical significance of these changes. Overall, clinicians perceived significant benefit to the patient.

Treatment was generally well tolerated, with few side effects or intolerances requiring changes to treatment. The average treatment duration was 30 months and 20 months for IBA and LEN, respectively, which reflects the long-term tolerability of both therapies. IBA administration [19] using intravenous push rather than intravenous infusion was the preferred route; LEN administration by subcutaneous injection every 6 months [22] provided convenience to patients, although there were several reports of injection site pain and/or nodules. Treatment failure was reported in 2/21 patients (9.5%).

Most patients had significant barriers to medication adherence including poor social or family support, comorbidity (eg, depression, anxiety, memory problems, complex medical histories), history of alcohol or substance use, unstable housing or financial support, pill fatigue with twice-daily dosing of oral meds, or dislike of medications as being a daily reminder of HIV. Four patients had previous gastrointestinal intolerance to oral medications, difficulty with swallowing, or required feeding tube for administration of oral medications. Only 1 patient initiated IBA + LEN because of a potential DDI. For those patients with adherence issues, parenteral administration of ARV can permit direct monitoring of medication use and regular follow-up to help provide better support. Some clinicians felt it was important to remove the burden of medication access and daily administration from patients.

Some patients benefitted from frequent touchpoints with their treatment team and became more invested in their treatment and changes to viral load or CD4^+^ count. The preference to receive IBA injections at home or at the clinic depended on patient factors such as insurance coverage, scheduling, confidentiality, and trust. Those who were reluctant to share their diagnosis with family or housemates preferred to receive treatments at the clinic.

Other case series of PWH using injectable-only regimens because of adherence challenges or underlying resistance have noted similarities to this population. A published case series of 34 PWH using LEN + CAB (± RPV) reported adherence issues related to housing insecurity and substance abuse as well as a high baseline incidence of NNRTI mutations. The group was able to achieve high levels of viral suppression with this injectable-only combination [23].

Several limitations of this case series are related to the retrospective nature of the data collection. Not every clinician uses the same approach for monitoring VL and CD4^+^ count after switching ARVs. For this reason, we were only able to evaluate VLs obtained within 12 to 24 weeks (and CD4^+^ counts within 4 to 44 weeks) after the initiation of combined IBA + LEN. It would be preferable to obtain follow-up measurements during more discrete time periods and include sequential measurements over time. Similarly, the inclusion of patients with less than 6 months of IBA + LEN treatment would provide more information on early tolerability and remove some of the selection bias that this exclusion criterion may have introduced. Resistance testing for IBA and LEN was not commercially available [18] and therefore could not be tested in all patients with VL >400 copies/mL at follow-up. Summaries of resistance testing did not include documentation of International Antiviral Society-United States of America–designated mutations, and there may have been differences in how clinicians interpreted resistance testing in their clinical practice. Finally, not all side effects may be represented here given limitations of a retrospective study, inadequate documentation, and lack of data if the drugs were administered at an offsite location, like an injection clinic. Overall, data for a group of 21 patients is still helpful in assessing overall trends in effectiveness and to continue monitoring for safety and tolerability. It is hopeful that real-world studies and registry data will add to the evidence for combined IBA + LEN usage.

## CONCLUSION

The case series reinforces the complexity of treating the HTE MDR HIV priority population—they often have many unmet needs, extensive histories of ARV use, and barriers to adherence. It is important to understand individual needs and preferences and to provide treatment options that address these important factors. Combined use of IBA + LEN ± OBR is 1 such option. It was well tolerated and resulted in clinically significant changes to VL, as well as improvements in CD4^+^ count in this small cohort of HTE MDR PWH.

## Supplementary Material

ciaf597_Supplementary_Data

## Supplementary Data

Supplementary materials are available at *Clinical Infectious Diseases* online. Consisting of data provided by the authors to benefit the reader, the posted materials are not copyedited and are the sole responsibility of the authors, so questions or comments should be addressed to the corresponding author.

## References

[1] Custer B, Altan E, Montalvo L, et al HIV subtypes and drug-resistance-associated mutations in US blood donors, 2015–2020. Open Forum Infect Dis 2024; 11:ofae343. 38994445 10.1093/ofid/ofae343 PMC11237352

[2] McClung RP, Oster AM, Ocfemia MCB, et al Transmitted drug resistance among human immunodeficiency virus (HIV)-1 diagnoses in the United States, 2014–2018. Clin Infect Dis 2022; 74:1055–62. 34175948 10.1093/cid/ciab583 PMC9630858

[3] Andreatta K, D’Antoni ML, Chang S, et al High efficacy of bictegravir/emtricitabine/tenofovir alafenamide (B/F/TAF) in Black adults in the United States, including those with pre-existing HIV resistance and suboptimal adherence. J Med Virol 2024; 96:e2899. 10.1002/jmv.29899 39370775

[4] WHO Guidelines Approved by the Guidelines Review Committee . Consolidated guidelines on HIV prevention, testing, treatment, service delivery and monitoring: recommendations for a public health approach. Geneva, Switzerland: World Health Organization, 2021. 34370423

[5] U.S. Department of Health and Human Services. Guidelines for the Use of Antiretroviral Agents in Adults and Adolescents with HIV [updated 2024]. Available at: https://clinicalinfo.hiv.gov/en/guidelines/adult-and-adolescent-arv. Accessed 11 November 2025.

[6] Armenia D, Di Carlo D, Flandre P, et al HIV MDR is still a relevant issue despite its dramatic drop over the years. J Antimicrob Chemother 2020; 75:1301–10. 31976521 10.1093/jac/dkz554

[7] Zaccarelli M, Tozzi V, Lorenzini P, et al Multiple drug class-wide resistance associated with poorer survival after treatment failure in a cohort of HIV-infected patients. AIDS 2005; 19:1081–9. 15958840 10.1097/01.aids.0000174455.01369.ad

[8] European AIDS Clinical Society. EACS Guidelines version 12.1, November 2024. Available at: https://eacs.sanfordguide.com. Accessed 11 November 2025.

[9] Menza TW, Hixson LK, Lipira L, Drach L. Social determinants of health and care outcomes among people with HIV in the United States. Open Forum Infect Dis 2021; 8:ofab330. 34307729 10.1093/ofid/ofab330 PMC8297699

[10] Moretti M, Stoffels K, Van Laethem K, Verhofstede C, Van Den Wijngaert S, Martin C. The challenge of adherence to a complex antiretroviral therapy regimen in an individual with multidrug-resistant HIV. Top Antivir Med 2024; 32:437–44. 39141922 PMC11293606

[11] Orkin C, Cahn P, Castagna A, et al Opening the door on entry inhibitors in HIV: redefining the use of entry inhibitors in heavily treatment experienced and treatment-limited individuals living with HIV. HIV Med 2022; 23:936–46. 35293094 10.1111/hiv.13288 PMC9546304

[12] Aberg JA, Shepherd B, Wang M, et al Week 240 efficacy and safety of fostemsavir plus optimized background therapy in heavily treatment-experienced adults with HIV-1. Infect Dis Ther 2023; 12:2321–35. 37751019 10.1007/s40121-023-00870-6 PMC10581994

[13] Philbin MM, Parish CL, Kinnard EN, et al Multisite study of women living with HIV's perceived barriers to, and interest in, long-acting injectable antiretroviral therapy. J Acquir Immune Defic Syndr 2020; 84:263–70. 32530905 10.1097/QAI.0000000000002337 PMC7483266

[14] Konishi K, Onozuka D, Okubo M, Kasamatsu Y, Kutsuna S, Shirano M. Long-acting antiretroviral therapy effectiveness and patient satisfaction using patient questionnaires: data from a real-world setting. BMC Infect Dis 2024; 24:979. 39278923 10.1186/s12879-024-09904-x PMC11404019

[15] Kilcrease C, Yusuf H, Park J, et al Realizing the promise of long-acting antiretroviral treatment strategies for individuals with HIV and adherence challenges: an illustrative case series. AIDS Res Ther 2022; 19:56. 36435793 10.1186/s12981-022-00477-w PMC9701425

[16] John M, Williams L, Nolan G, Bonnett M, Castley A, Nolan D. Real-world use of long-acting cabotegravir and rilpivirine: 12-month results of the inJectable Antiretroviral therapy feasiBility Study (JABS). HIV Med 2024; 25:935–45. 38644518 10.1111/hiv.13647

[17] Orkin C, Schapiro JM, Perno CF, et al Expanded multivariable models to assist patient selection for long-acting cabotegravir + rilpivirine treatment: clinical utility of a combination of patient, drug concentration, and viral factors associated with virologic failure. Clin Infect Dis 2023; 77:1423–31. 37340869 10.1093/cid/ciad370 PMC10654860

[18] Cluck DB, Chastain DB, Murray M, et al Consensus recommendations for the use of novel antiretrovirals in persons with HIV who are heavily treatment-experienced and/or have multidrug-resistant HIV-1: endorsed by the American Academy of HIV Medicine, American College of Clinical Pharmacy: an executive summary. Pharmacotherapy 2024; 44:354–9. 38853605 10.1002/phar.2913

[19] Emu B, Fessel J, Schrader S, et al Phase 3 study of ibalizumab for multidrug-resistant HIV-1. N Engl J Med 2018; 379:645–54. 30110589 10.1056/NEJMoa1711460

[20] TROGARZO (ibalizumab-uiyk) prescribing information. Theratechnologies Inc., Montreal, QC, Canada. Revised December 2023. Available at: https://www.accessdata.fda.gov/drugsatfda_docs/label/2023/761065s020lbl.pdf. Accessed 11 November 2025.

[21] Segal-Maurer S, DeJesus E, Stellbrink HJ, et al Capsid inhibition with lenacapavir in multidrug-resistant HIV-1 infection. N Engl J Med 2022; 386:1793–803. 35544387 10.1056/NEJMoa2115542

[22] SUNLECA (lenacapavir) prescribing information. Gilead Sciences Inc., Foster City, CA, USA. Revised November 2024. Available at: https://www.accessdata.fda.gov/drugsatfda_docs/label/2024/215973s006,215974s008lbl.pdf. Accessed 11 November 2025.

[23] Gandhi M, Hill L, Grochowski J, et al Case series of people with HIV on the long-acting combination of lenacapavir and cabotegravir: call for a trial. Open Forum Infect Dis 2024; 11:ofae125. 38628952 10.1093/ofid/ofae125 PMC11020301

