# Supplementary material for: Combined Use of Ibalizumab and Lenacapavir for the Treatment of Multidrug-resistant HIV-1: A Case Series
Source: Clin Infect Dis. 2025 Nov 3;82(3):e534–40. doi: 10.1093/cid/ciaf597 (PMC13017887; doi:10.1093/cid/ciaf597)
Supplement: ciaf597_Supplementary_Data [file ciaf597_supplementary_data.docx]

**SUPPLEMENTAL**

**Supplementary Table 1. Clinical data summary**

| **Patient number** | **1** | **2** | **4** | **5** | **7** |
| --- | --- | --- | --- | --- | --- |
| **Sex** | Male | Female | Male | Male | Male |
| **Race and/or ethnicity** | White | African American non-Hispanic | White non-Hispanic | African American | African American, Hispanic |
| **Current age (years)** | 51 | 30 | 59 | 42 | 66 |
| **Age at HIV-1 diagnosis** | 23 | 27 | 21 | 30 | 29 |
| **Relevant medical history** | Tongue squamous cell cancer stage IV (metastatic to lungs) | Stomatitis Acute Esophagitis Oral Thrush Folliculitis | CAD, Anxiety, Peripheral polyneuropathy, Depression, Chronic Leukopenia, Cancer of rectum, anus, and anal canal, Genital herpes, Hx of non-Hodgkin’s Lymphoma, HTN, Asthma, Chronic pain syndrome, Anorexia | Esophageal Disease with Candida Chronic Pain – accident resulted in several fractures and surgeries Hypertension  Dental Caries – long history of dental pain and infections | Previous HCV HBV CVA Hypogonadism CKD Fatty liver with hepatomegaly Herpes simplex Dementia and memory loss |
|  |  |  |  |  |  |
| **ARV regimen just prior to ibalizumab (IBA)/lenacapavir (LEN) initiation** | RTV/TPV + FTC/TAF | CAB + DRV/COBI/FTC/TAF | RTV + FTC/TAF + DRV + DTG + DOR | DTG + DRV/COBI/FTC/TAF | BIC/FTC/TAF + ETR + FTR |
| **Summary of resistance testing** | NRTI: Resistance NNRTI: Resistance PI: Resistance INSTI: Resistance | NRTI: Resistance NNRTI: - PI: - INSTI: - | NRTI: Resistance  NNRTI: - PI: Resistance  INSTI: - | NRTI: Resistance NNRTI: Resistance  PI: Resistance  INSTI: - | NRTI: Resistance NNRTI: Resistance PI: Resistance INSTI: Resistance |
| **Baseline HIV-1 viral load prior to starting IBA+LEN (copies/mL)** | 50 | 7,210 | 254 | 343,000 | 258 |
| **Follow-up VL (copies/mL)** | <20 | <20 | 806 | 24 | <50 |
| **Baseline CD4^+^ count prior to starting IBA+LEN (cells/mm^3^)** | 364 | 136 | 257 | <32 | 330 |
| **Follow-up CD4^+^ count (cells/mm^3^)** | 295 | 353 | 282 | 183 | 226 |
| **Administration of IBA and LEN** | IBA home; LEN by clinician in the office | IBA and LEN: clinic | IBA home and LEN clinic | IBA home and LEN clinic | IBA and LEN at clinician's office |
| **Optimized background regimen (OBR) with IBA and LEN** | 3TC + FTR | CAB/RPV | RTV + DRV + DOR  + (FTC/TAF + DTG later changed to BIC/FTC/TAF) | None | FTR |
| **Duration of IBA therapy** | 18 months | 15 months | 32 months | 18 months | 20 months |
| **Duration of LEN therapy** | 18 months | 16 months | 56 months | 18 months | 19 months |
| **Were there any changes to OBR during the treatment period?** | No | No | Regimen change: Yes, FTC/TAF + DTG were discontinued to start BIC/FTC/TAF | n/a |  |

3TC, lamivudine; ABC, abacavir; ARV, antiretroviral; ATZ, atazanavir; AV, arteriovenous; AZT, zidovudine; BIC, bictegravir; BID, twice daily; CAB, cabotegravir; CAD, coronary artery disease; CKD, chronic kidney disease; COPD, chronic obstructive pulmonary disease; CVA, cerebrovascular accident; DRV, darunavir; DTG, dolutegravir; DVT, deep vein thrombosis; ED, erectile dysfunction; EFV, efavirenz; ETR, etravirine; FTC, emtricitabine; FTR, fostemsavir; GERD, gastroesophageal reflux disease; HBV, hepatitis B virus; HCV, hepatitis C virus; HPV, human papilloma virus; HSV, herpes simplex virus; HTN, hypertension; Hx, history; IBA, ibalizumab; INSTI, integrase strand transfer inhibitor; LA, long-acting; LEN, lenacapavir; MVC, maraviroc; MSSA, methicillin-sensitive *S. aureus*; NFV, nelfinavir; NNRTI, non-nucleoside reverse transcriptase inhibitor; NRTI, nucleoside reverse transcriptase inhibitor; NVP, nevirapine; OBR, optimized background regimen; PCP, pneumocystis pneumonia; PE, pulmonary embolism; PI, protease inhibitor; PRO 140, leronlimab; PTSD, post-traumatic stress disorder; RAL, raltegravir; RPV, rilpirivine; RTV, ritonavir; SQV, saquinavir; T2D, type 2 diabetes; T-20, enfurvirtide; TAF/TDF, tenofovir; VL, viral load; ZDV, zidovudine.

| **Patient number** | **8** | **9** | **10** | **11** | **12** |
| --- | --- | --- | --- | --- | --- |
| **Sex** | Female | Male | Male | Male | Male |
| **Race and/or ethnicity** | African American | White non-Hispanic | Caucasian | African American non-Hispanic | African American |
| **Current age (years)** | 54 | 69 | 64 | 34 | 70 |
| **Age at HIV-1 diagnosis** | 30 | 34 | 29 | 8 | 30 |
| **Relevant medical history** | Vulval intraepithelial neoplasia  Genital Herpes Simplex  Hypertension  Anemia  Chronic Headaches | Oral thrush and mouth ulcer, HTN, CAD, Aneurysm ascending aorta, GERD, CKD, Dyslipidemia, Grave’s disease, Chronic pain and headache, Depression and Anxiety, Insomnia, Panic Attack, Hypogonadism, Peripheral Neuropathy, Granuloma of liver, Lipodystrophy, Vitamin D Deficiency, BPH, Hyperthyroidism | CVA Deep Venous Thrombosis  Depression  Hyperlipidemia | Herpes simplex-2, Uveitis, Steven-Johnson syndrome, MSSA, Blood glucose abnormal, Candida esophagitis, Drug-Induced liver injury, Asthma | CKD-3B with right AV graft, hemodialysis  Depression (in remission)  Hypertension  Sinus tachycardia  COPD  Nuclear sclerosis of both eyes  Chronic pain and sciatica |
|  |  |  |  |  |  |
| **ARV regimen just prior to ibalizumab (IBA)/lenacapavir (LEN) initiation** | DRV/COBI/FTC/TAF | DRV + RTV + RAL + IBA | DTG+DRV+RTV | BIC/FTC/TAF | BIC/FTC/TAF + IBA + FTR |
| **Summary of resistance testing** | NRTI: Resistance  NNRTI: -  PI: -  INSTI: Resistance | NRTI: Resistance  NNRTI: Resistance  PI: Resistance  INSTI: - | NRTI: Resistance  NNRTI: Resistance  PI: Resistance  INSTI: Resistance | NRTI: Resistance  NNRTI: -  PI: -  INSTI: Probable resistance | NRTI: Resistance  NNRTI: Resistance  PI: Resistance  INSTI: Resistance |
| **Baseline HIV-1 viral load prior to starting IBA+LEN (copies/mL)** | 1,200,000 | <20 | <50 | 744,000 | 2,930 |
| **Follow-up VL (copies/mL)** | 123 | <20 | <50 | 32 | 220 |
| **Baseline CD4^+^ count prior to starting IBA+LEN (cells/mm^3^)** | <32 | 361 | 539 | 29 | 298 |
| **Follow-up CD4^+^ count (cells/mm^3^)** | 100 | 399 | 523 | 106 | 192 |
| **Administration of IBA and LEN** | IBA home and LEN clinic | IBA home and LEN clinic | IBA home and LEN clinic | IBA home and LEN clinic | IBA home and LEN home |
| **Optimized background regimen (OBR) with IBA and LEN** | None | DRV + RTV + RAL | None | CAB/RPV | FTR |
| **Duration of IBA therapy** | 18 months | 70 months | 12 months | 15 months | 72 months |
| **Duration of LEN therapy** | 18 months | 10 months | 20 months | 14 months | 19 months |
| **Were there any changes to OBR during the treatment period?** | No.  Treatment failure; IBA + LEN discontinued* | Simplified: DRV + RTV + RAL + IBA -> RAL + IBA, then + LEN | No.  IBA discontinued due to suspected adverse effect (clotting) | No | Drug added: Patient was viremic with VL> 1400 on FTR + IBA + LEN; added 3TC |

*Follow up resistance testing indicated the following mutations: CA: I6L, I15L, V27I, S41A/T, Q67H, K70R, A77A/V, V83L, I91V, A105A/T, N120N/S,T148V, A177C, S178T, E180D.
3TC, lamivudine; ABC, abacavir; ARV, antiretroviral; ATZ, atazanavir; AV, arteriovenous; AZT, zidovudine; BIC, bictegravir; BID, twice daily; CAB, cabotegravir; CAD, coronary artery disease; CKD, chronic kidney disease; COPD, chronic obstructive pulmonary disease; CVA, cerebrovascular accident; DRV, darunavir; DTG, dolutegravir; DVT, deep vein thrombosis; ED, erectile dysfunction; EFV, efavirenz; ETR, etravirine; FTC, emtricitabine; FTR, fostemsavir; GERD, gastroesophageal reflux disease; HBV, hepatitis B virus; HCV, hepatitis C virus; HPV, human papilloma virus; HSV, herpes simplex virus; HTN, hypertension; Hx, history; IBA, ibalizumab; INSTI, integrase strand transfer inhibitor; LA, long-acting; LEN, lenacapavir; MVC, maraviroc; MSSA, methicillin-sensitive *S. aureus*; NFV, nelfinavir; NNRTI, non-nucleoside reverse transcriptase inhibitor; NRTI, nucleoside reverse transcriptase inhibitor; NVP, nevirapine; OBR, optimized background regimen; PCP, pneumocystis pneumonia; PE, pulmonary embolism; PI, protease inhibitor; PRO 140, leronlimab; PTSD, post-traumatic stress disorder; RAL, raltegravir; RPV, rilpirivine; RTV, ritonavir; SQV, saquinavir; T2D, type 2 diabetes; T-20, enfurvirtide; TAF/TDF, tenofovir; VL, viral load; ZDV, zidovudine.

| **Patient number** | **13** | **14** | **15** | **16** | **19** |
| --- | --- | --- | --- | --- | --- |
| **Sex** | Female | Male | Male | Male | Male |
| **Race and/or ethnicity** | African American | White non-Hispanic | White Hispanic | African American | White  Non-Hispanic |
| **Current age (years)** | 29 | 59 | 31 | Not specified | 60 |
| **Age at HIV-1 diagnosis** | At birth | 20 | At birth | 46 | 26 |
| **Relevant medical history** | PCP  Hearing loss from ear infection as a child (cochlear implant)  Depression  PTSD  Anxiety | CAD  Obesity  Hypogonadism  Hyperlipidemia  Anemia  Elevated fasting glucose  Fatigue  Anal Cancer  HTN  HSV | Learning disabilities  Herpes Simplex  Pilonidal cyst | Depression  Pneumonia  Oral candidiasis  Depression  Severe noncompliance with HIV medications  HIV dementia | Hypogonadism, T2D, Prostate Cancer, Dyslipidemia, Hypothyroidism, ED, B12 Deficiency, Anxiety, Gynecomastia, Primary cancer of skin of hand, Herpes, COPD, Shortness of breath, Anal lesion, Adrenal insufficiency |
|  |  |  |  |  |  |
| **ARV regimen just prior to ibalizumab (IBA)/lenacapavir (LEN) initiation** | DRV/COBI/FTC/TAF | Pre-IBA: BIC/FTC/TAF + ETR  Pre-LEN: BIC/FTC/TAF + FTR + DOR + IBA | Pre-IBA: RTV +DTG + DRV + FTC/TAF  Pre-LEN: BIC/FTC/TAF + DOR + T-30 + IBA | TVF + DTG + DRV/COBI | BIC/FTC/TAF + DOR + FTR |
| **Summary of resistance testing** | NRTI: Resistance  NNRTI: Resistance  PI: -  INSTI: - | NRTI: Resistance  NNRTI: Resistance  PI: Resistance  INSTI: Resistance | NRTI: Resistance  NNRTI: Resistance  PI: Resistance  INSTI: Resistance | NRTI: Resistance  NNRTI: -  PI: Resistance  INSTI: Resistance | NRTI: Resistance  NNRTI: Resistance  PI: Resistance  INSTI: Resistance |
| **Baseline HIV-1 viral load prior to starting IBA+LEN (copies/mL)** | 13,700 | 171 | 8,540 | 228,000 | 51,900 |
| **Follow-up VL (copies/mL)** | 4,210 | 190 | 6,690 | <20 | <20 |
| **Baseline CD4^+^ count prior to starting IBA+LEN (cells/mm^3^)** | 137 | 65 | 327 | <20 | 600 |
| **Follow-up CD4^+^ count (cells/mm^3^)** | 204 | Not evaluable | Not evaluable | 83 | 875 |
| **Administration of IBA and LEN** | IBA: Infusion clinic  LEN: Clinic | IBA home and LEN clinic | IBA home and LEN clinic | IBA: Infusion clinic  LEN: Not specified | IBA home and LEN clinic |
| **Optimized background regimen (OBR) with IBA and LEN** | None | BIC/FTC/TAF + DOR + FTR | BIC/FTC/TAF + DOR | TVF + CAB/RPV | BIC/FTC/TAF + T-30 |
| **Duration of IBA therapy** | 9 months | 60 months | 72 months | 32 months | 16 months |
| **Duration of LEN therapy** | 9 months | 12 months | 48 months | 32 months | 16 months |
| **Were there any changes to OBR during the treatment period?** | No.  Treatment failure; IBA + LEN discontinued | No | No | No | No, but will consider stopping IBA once the patient is undetectable for a longer period of time |

3TC, lamivudine; ABC, abacavir; ARV, antiretroviral; ATZ, atazanavir; AV, arteriovenous; AZT, zidovudine; BIC, bictegravir; BID, twice daily; CAB, cabotegravir; CAD, coronary artery disease; CKD, chronic kidney disease; COPD, chronic obstructive pulmonary disease; CVA, cerebrovascular accident; DRV, darunavir; DTG, dolutegravir; DVT, deep vein thrombosis; ED, erectile dysfunction; EFV, efavirenz; ETR, etravirine; FTC, emtricitabine; FTR, fostemsavir; GERD, gastroesophageal reflux disease; HBV, hepatitis B virus; HCV, hepatitis C virus; HPV, human papilloma virus; HSV, herpes simplex virus; HTN, hypertension; Hx, history; IBA, ibalizumab; INSTI, integrase strand transfer inhibitor; LA, long-acting; LEN, lenacapavir; MVC, maraviroc; MSSA, methicillin-sensitive *S. aureus*; NFV, nelfinavir; NNRTI, non-nucleoside reverse transcriptase inhibitor; NRTI, nucleoside reverse transcriptase inhibitor; NVP, nevirapine; OBR, optimized background regimen; PCP, pneumocystis pneumonia; PE, pulmonary embolism; PI, protease inhibitor; PRO 140, leronlimab; PTSD, post-traumatic stress disorder; RAL, raltegravir; RPV, rilpirivine; RTV, ritonavir; SQV, saquinavir; T2D, type 2 diabetes; T-20, enfurvirtide; TAF/TDF, tenofovir; VL, viral load; ZDV, zidovudine.

| **Patient number** | **20** | **23** | **24** | **25** | **26** |
| --- | --- | --- | --- | --- | --- |
| **Sex** | Male | Female | Female | Male | Male |
| **Race and/or ethnicity** | Black non-Hispanic | Race unknown Hispanic | African American | White Hispanic | Black |
| **Current age (years)** | 57 | 52 | 30 | 50 | 37 |
| **Age at HIV-1 diagnosis** | 32 | 18 | At birth | 19 | 24 |
| **Relevant medical history** | DVT/PE  CKD stage 3A2 | DVT/PE  Depression | Depression/ dysthymia; swallowing difficulty with G-tube used for meds | Chronic Kaposi's Sarcoma  Obesity | Lupus, Autoimmune Hepatitis/Cholangitis, Steroid-induced diabetes, Osteopenia, Cataracts, Neuropathy. Abdominal pain due to autoimmune disease affecting ability to take oral medications. |
|  |  |  |  |  |  |
| **ARV regimen just prior to ibalizumab (IBA)/lenacapavir (LEN) initiation** | DOR + DRV/COBI/TAF/FTC | IBA + DTG (BID) + FTR | DTG/ABC/3TC+ ATZ | DTG (BID) + 3TC + DRV/COBI | FTR + IBA + DTG + DOR |
| **Summary of resistance testing** | NRTI: Resistance to 3TC/FTC  NNRTI: Partial resistance to RPV, DOR  PI: -  INSTI: Partial resistance to RAL, BIC, EVG, DTG | NRTI: Resistance  NNRTI: -  PI: -  INSTI: Possible low level resistance to RAL | NRTI: Resistance  NNRTI: Resistance  PI: -  INSTI: Probable resistance | NRTI: Resistance  NNRTI: Resistance  PI: Resistance  INSTI: Resistance | Last resistance testing in 2012.  Since then, VL not high enough for testing |
| **Baseline HIV-1 viral load prior to starting IBA+LEN (copies/mL)** | 760,000 | <20 | 173 | 278 | 7,830 |
| **Follow-up VL (copies/mL)** | 80 | <20 | <20 | <20 | <20 |
| **Baseline CD4^+^ count prior to starting IBA+LEN (cells/mm^3^)** | 25 | 491 | 126 | 609 | 342 |
| **Follow-up CD4^+^ count (cells/mm^3^)** | 146 | 594 | 254 | 668 | Not evaluable |
| **Administration of IBA and LEN** | IBA at Infusion clinic and LEN at outpatient clinic | IBA at Infusion clinic and LEN at outpatient clinic | IBA at Infusion clinic and LEN at outpatient clinic | IBA at Infusion clinic and LEN at outpatient clinic | IBA and LEN: RN in clinic |
| **Optimized background regimen (OBR) with IBA and LEN** | CAB/RPV | CAB/RPV | DTG (BID)+ ABC/3TC | FTR + DOR | FTR + DTG + DOR |
| **Duration of IBA therapy** | 12 months | 18 months | 18 months | 20 months | 48 months |
| **Duration of LEN therapy** | 12 months | 12 months | 18 months | 20 months | 27 months |
| **Were there any changes to OBR during the treatment period?** | No | No | No | No | Yes.  Simplification: Stopped DTG |

3TC, lamivudine; ABC, abacavir; ARV, antiretroviral; ATZ, atazanavir; AV, arteriovenous; AZT, zidovudine; BIC, bictegravir; BID, twice daily; CAB, cabotegravir; CAD, coronary artery disease; CKD, chronic kidney disease; COPD, chronic obstructive pulmonary disease; CVA, cerebrovascular accident; DRV, darunavir; DTG, dolutegravir; DVT, deep vein thrombosis; ED, erectile dysfunction; EFV, efavirenz; ETR, etravirine; FTC, emtricitabine; FTR, fostemsavir; GERD, gastroesophageal reflux disease; HBV, hepatitis B virus; HCV, hepatitis C virus; HPV, human papilloma virus; HSV, herpes simplex virus; HTN, hypertension; Hx, history; IBA, ibalizumab; INSTI, integrase strand transfer inhibitor; LA, long-acting; LEN, lenacapavir; MVC, maraviroc; MSSA, methicillin-sensitive *S. aureus*; NFV, nelfinavir; NNRTI, non-nucleoside reverse transcriptase inhibitor; NRTI, nucleoside reverse transcriptase inhibitor; NVP, nevirapine; OBR, optimized background regimen; PCP, pneumocystis pneumonia; PE, pulmonary embolism; PI, protease inhibitor; PRO 140, leronlimab; PTSD, post-traumatic stress disorder; RAL, raltegravir; RPV, rilpirivine; RTV, ritonavir; SQV, saquinavir; T2D, type 2 diabetes; T-20, enfurvirtide; TAF/TDF, tenofovir; VL, viral load; ZDV, zidovudine.

| **Patient number** | **27** |
| --- | --- |
| **Sex** | Female |
| **Race and/or ethnicity** | Black |
| **Current age (years)** | 74 |
| **Age at HIV-1 diagnosis** | 55 |
| **Relevant medical history** | Heart failure, DVT, HTN, Hyperlipidemia, COPD, Pulmonary HTN, Vulvar Dysplasia, CKD stage 2, Osteoarthritis. Patient has had chronic difficulty in swallowing pills (crushes pills) and has had virologic failure due to that. |
|  |  |
| **ARV regimen just prior to ibalizumab (IBA)/lenacapavir (LEN) initiation** | 3TC, IBA, LA CAB/RPV immediately pre-LEN |
| **Summary of resistance testing** | NRTI: -  NNRTI: Resistance  PI: -  INSTI: Resistance |
| **Baseline HIV-1 viral load prior to starting IBA+LEN (copies/mL)** | 5,350 |
| **Follow-up VL (copies/mL)** | <20 |
| **Baseline CD4^+^ count prior to starting IBA+LEN (cells/mm^3^)** | 189 |
| **Follow-up CD4^+^ count (cells/mm^3^)** | 420 |
| **Administration of IBA and LEN** | Initially IBA was infused at home then switched to IV push at clinic.  LEN always administered by clinic RN staff. |
| **Optimized background regimen (OBR) with IBA and LEN** | DRV/rit, 3TC |
| **Duration of IBA therapy** | 34 months |
| **Duration of LEN therapy** | 14 months |
| **Were there any changes to OBR during the treatment period?** | No |

3TC, lamivudine; ABC, abacavir; ARV, antiretroviral; ATZ, atazanavir; AV, arteriovenous; AZT, zidovudine; BIC, bictegravir; BID, twice daily; CAB, cabotegravir; CAD, coronary artery disease; CKD, chronic kidney disease; COPD, chronic obstructive pulmonary disease; CVA, cerebrovascular accident; DRV, darunavir; DTG, dolutegravir; DVT, deep vein thrombosis; ED, erectile dysfunction; EFV, efavirenz; ETR, etravirine; FTC, emtricitabine; FTR, fostemsavir; GERD, gastroesophageal reflux disease; HBV, hepatitis B virus; HCV, hepatitis C virus; HPV, human papilloma virus; HSV, herpes simplex virus; HTN, hypertension; Hx, history; IBA, ibalizumab; INSTI, integrase strand transfer inhibitor; LA, long-acting; LEN, lenacapavir; MVC, maraviroc; MSSA, methicillin-sensitive *S. aureus*; NFV, nelfinavir; NNRTI, non-nucleoside reverse transcriptase inhibitor; NRTI, nucleoside reverse transcriptase inhibitor; NVP, nevirapine; OBR, optimized background regimen; PCP, pneumocystis pneumonia; PE, pulmonary embolism; PI, protease inhibitor; PRO 140, leronlimab; PTSD, post-traumatic stress disorder; RAL, raltegravir; RPV, rilpirivine; RTV, ritonavir; SQV, saquinavir; T2D, type 2 diabetes; T-20, enfurvirtide; TAF/TDF, tenofovir; VL, viral load; ZDV, zidovudine.

**IRB exemption**

**
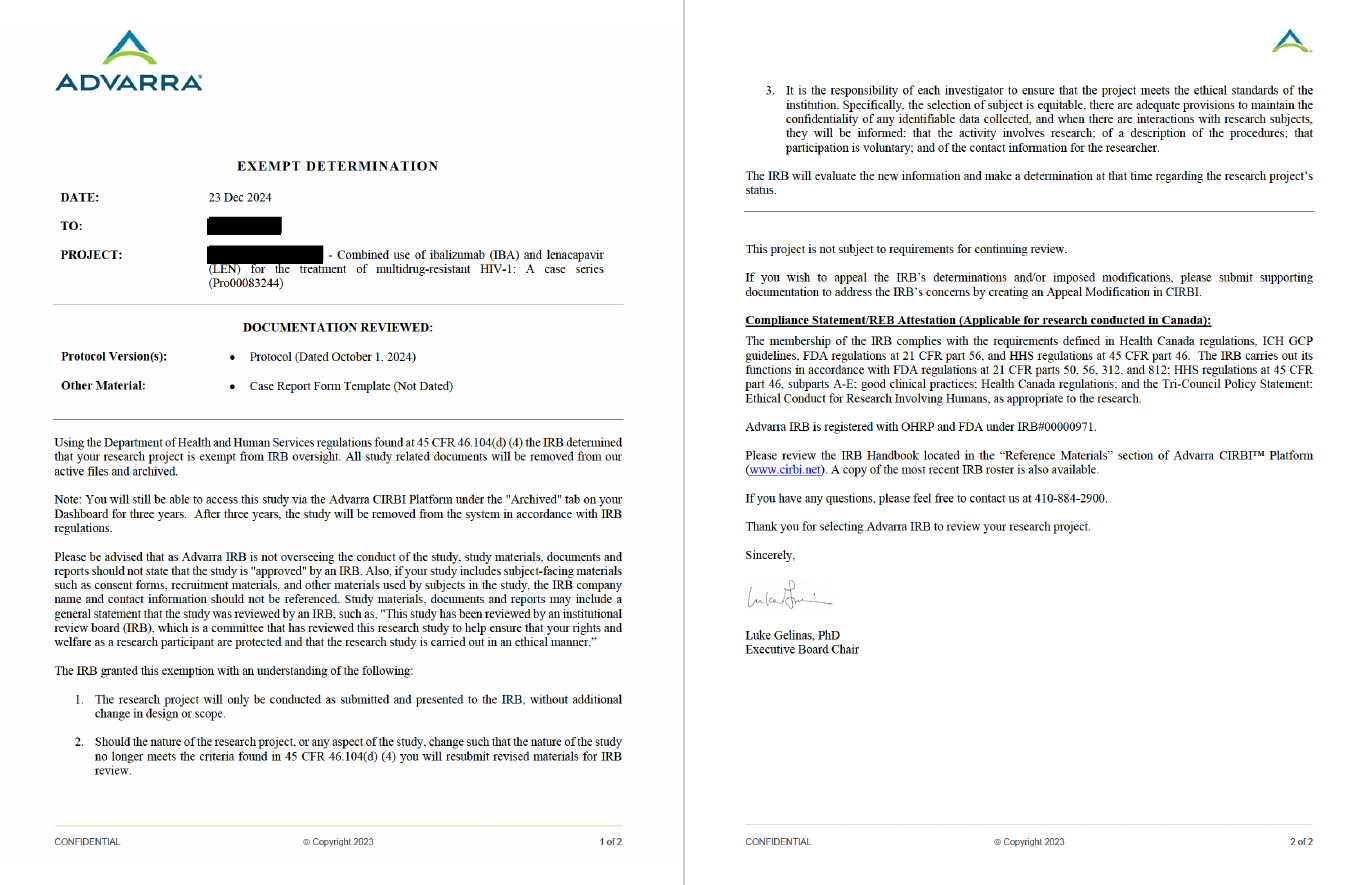
**

**Case collection form**

**
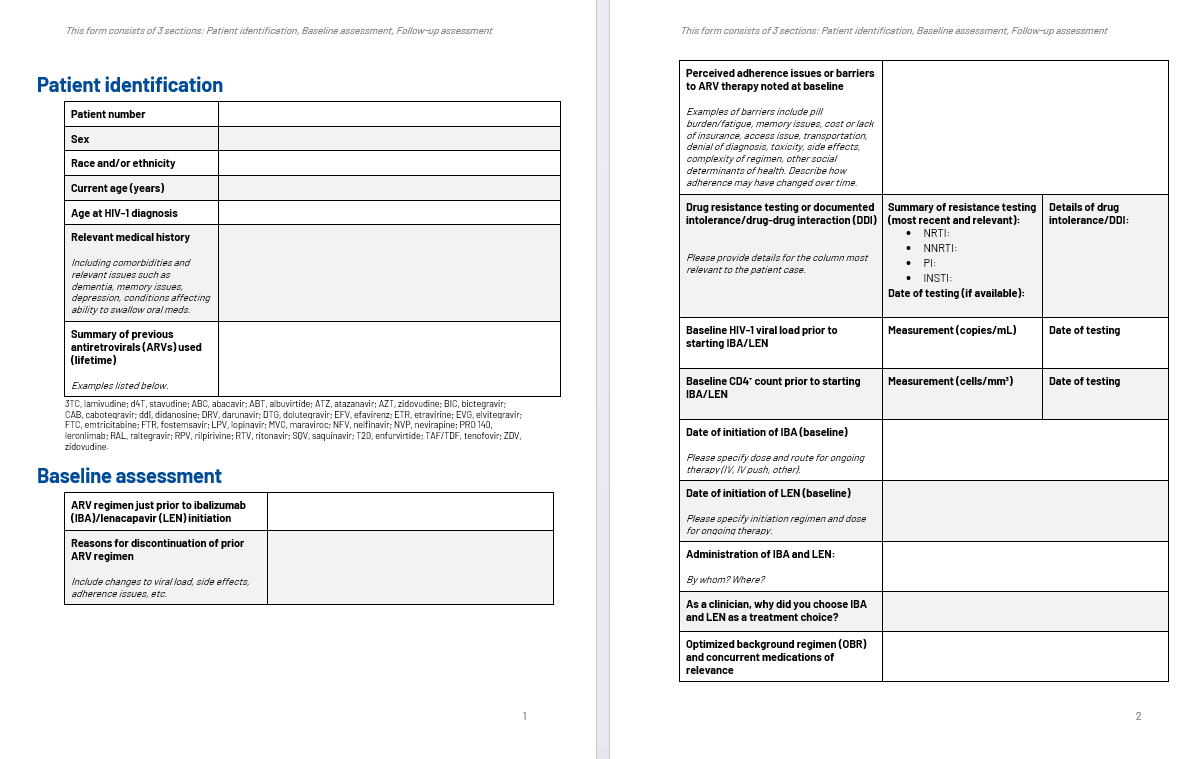
**

**
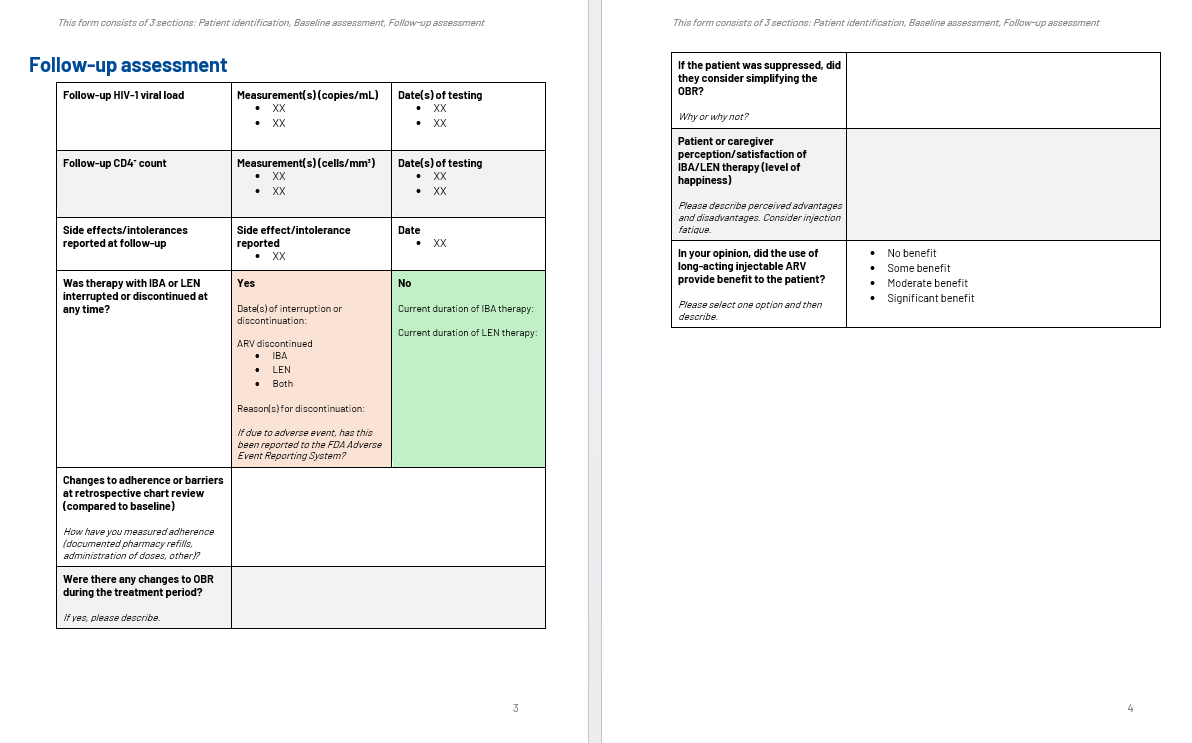
**
